# Field-Theoretic Simulations for Block Copolymer Melts Using the Partial Saddle-Point Approximation

**DOI:** 10.3390/polym13152437

**Published:** 2021-07-24

**Authors:** Mark W. Matsen, Thomas M. Beardsley

**Affiliations:** 1 Department of Chemical Engineering, University of Waterloo, Waterloo, ON N2L 3G1, Canada; 2 Department of Physics & Astronomy, University of Waterloo, Waterloo, ON N2L 3G1, Canada; 3 Waterloo Institute for Nanotechnology, University of Waterloo, Waterloo, ON N2L 3G1, Canada; tbeardsl@uwaterloo.ca

**Keywords:** block copolymer melts, field-theoretic simulations, molecular self-assembly, phase diagrams, order-disorder transitions, gyroid phase, Fddd phase, bicontinuous microemulsion

## Abstract

Field-theoretic simulations (FTS) provide an efficient technique for investigating fluctuation effects in block copolymer melts with numerous advantages over traditional particle-based simulations. For systems involving two components (i.e., A and B), the field-based Hamiltonian, $H_{f}\left[W_{-},W_{+}\right]$, depends on a composition field, $W_{-}\left(r\right)$, that controls the segregation of the unlike components and a pressure field, $W_{+}\left(r\right)$, that enforces incompressibility. This review introduces researchers to a promising variant of FTS, in which $W_{-}\left(r\right)$ fluctuates while $W_{+}\left(r\right)$ tracks its mean-field value. The method is described in detail for melts of AB diblock copolymer, covering its theoretical foundation through to its numerical implementation. We then illustrate its application for neat AB diblock copolymer melts, as well as ternary blends of AB diblock copolymer with its A- and B-type parent homopolymers. The review concludes by discussing the future outlook. To help researchers adopt the method, open-source code is provided that can be run on either central processing units (CPUs) or graphics processing units (GPUs).

## 1. Introduction

Block copolymers refer to polymeric molecules composed of two chemically-distinct segments, generally denoted as A and B, that are grouped together into separate sections or rather blocks [1,2]. The simplest and most common is the AB diblock copolymer, a linear chain of $N=N_{A}+N_{B}$ segments in which the first $N_{A}$ are of type A and the last $N_{B}$ are of type B. Here, we follow the common practice of defining A and B segments such that they occupy equal-sized volumes, $\rho_{0}^{-1}$. The segments will nevertheless have different statistical lengths, $a_{A}$ and $a_{B}$, such that the natural end-to-end length of the entire molecule is $R_{0}=a_{A}N_{A}^{1/2}+a_{B}N_{B}^{1/2}$. For convenience, we define an average segment length *a* such that $R_{0}=aN^{1/2}$. The segments will also differ in their interactions, usually resulting in an incompatibility characterized by a positive Flory–Huggins interaction parameter, χ. If the product χN is sufficiently large, then the A and B components will microphase segregate into a periodically ordered morphology with domain sizes of order $R_{0}$.

The behavior of block copolymer melts is greatly simplified by the fact that it becomes universal in the limit of high molecular weight [3,4]. As a result, diblock copolymer melts are controlled by just four parameters: the segregation χN, the composition $f\equiv N_{A}/N$, the ratio of segment lengths $a_{A}/a_{B}$, and the invariant polymerization index $\bar{N}\equiv a^{6}\rho_{0}^{2}N$. None of the other details of the system have any impact on the coarse-grained behavior. This is true for experimental systems as well as theoretical models so long as they include the essential physics. Consequently, they can all be mapped onto the standard Gaussian chain model (GCM) [5], which is a minimal model that treats block copolymer systems as incompressible melts of thin elastic threads that interact by pairwise contact forces. At present, the most accurate method of performing the mapping is the Morse calibration [4,6], which has been well demonstrated for experiments [7] and particle-based simulations [8,9,10,11,12], as well as field-theoretic simulations [13].

The mathematical form of the GCM is ideally suited to polymer field theory, whereby the particle-based Hamiltonian is converted to a field-based Hamiltonian expressed in terms of fields rather than particle coordinates [14,15,16,17,18]. For a two-component system, there are two fields, $W_{A}\left(r\right)$ and $W_{B}\left(r\right)$, that act on A and B segments, respectively. However, in this case, it is generally more convenient to express the field-based Hamiltonian, $H_{f}\left[W_{-},W_{+}\right]$, in terms of a composition field $W_{-}\left(r\right)\equiv \left[W_{A}\left(r\right)-W_{B}\left(r\right)\right]/2$ and a pressure field $W_{+}\left(r\right)\equiv \left[W_{A}\left(r\right)+W_{B}\left(r\right)\right]/2$. The equilibrium behavior is then obtained by performing statistical mechanics, where the partition function involves functional integrals over the two fields.

The self-consistent field theory (SCFT) of Edwards [19] evaluates the functional integrals using the saddle-point approximation, which equates to mean-field theory. Helfand [20] was the first to apply SCFT to block copolymers in 1975. In this approximation, the free energy of a melt is given by $F=H_{f}\left[w_{-},w_{+}\right]$, where $w_{-}\left(r\right)$ and $w_{+}\left(r\right)$ denote the saddle-point of the Hamiltonian obtained by solving the self-consistent conditions $DH_{f}/DW_{-}=DH_{f}/DW_{+}=0$. SCFT has been extraordinarily successful in predicting the relative stability of well ordered phases, but not so successful near the order-disorder transition (ODT). For instance, SCFT predicts that the weakly ordered phases converge to a critical point [21], which occurs at f=0.5 and χN=10.495 for conformationally symmetric diblock copolymers [22]. As a result, the ODT is bounded by the spherical phase, whereas in experiments there is no critical point and instead all the ordered phases generally extend to the ODT [23]. This qualitative shortcoming of SCFT occurs because it neglects composition fluctuations, which are particularly important in the disordered phase [18].

The first correction for fluctuation effects was by Fredrickson and Helfand in 1987 [24]. They showed that SCFT corresponds to infinite $\bar{N}$ and that, for finite values characteristic of experiments (i.e., $10^{2}≲\bar{N}≲10^{4}$ [23]), the stability of the disordered phase is significantly enhanced. This shifts the ODT to higher χN, wiping out the critical point and creating direct transitions with the classical lamellar and cylindrical phases. Subsequent calculations [25] have shown that it also creates direct transitions between the complex gyroid phase and the disordered phase, in agreement with experiments [23]. On the other hand, calculations [26] have predicted that the fluctuations destabilize the complex Fddd phase, which is contrary to experiments [27,28]. This is not surprising given the significant approximations involved in the Fredrickson–Helfand theory, which also cause the spherical phase to become unstable at experimentally relevant values of $\bar{N}$. To deal with these inaccuracies, there have been efforts to develop a rigorous perturbation theory expressed as a diagrammatic expansion. This has culminated in the renormalized one-loop calculation (ROL) [29], which now provides an accurate treatment of the disordered phase. In particular, it provides our best prediction for the disordered-state structure function, S(k) [30,31], which is used in the Morse calibration to determine an effective χ parameter. Unfortunately though, the extension of ROL to ordered phases is seriously difficult.

An alternative is to simulate the field-based Hamiltonian. Ganesan and Fredrickson began exploring this possibility in 2001 [32]. One complication with the field-theoretic approach is that the integration of $W_{+}\left(r\right)$ is along the imaginary axis, which leads to a complex-valued $H_{f}\left[W_{-},W_{+}\right]$ and thus a Boltzmann weight that is not positive definite. As a consequence, standard simulation methods cannot be applied. Fredrickson and co-workers have dealt with this by performing complex-Langevin simulations [33,34]. However, unlike for conventional Langevin simulations, the trajectories in phase space are not guaranteed to be stable [14]. Indeed, complex-Langevin simulations have encountered instabilities [35,36], which initially limited simulations [33] to values of $\bar{N}$ well above the experimentally accessible range. More recent simulations [34], however, have managed to reduce the instability by relaxing the incompressibility constraint. Although this allowed simulations at a more realistic $\bar{N}\approx 10^{4}$, the resulting phase diagram lacked both a direct gyroid-to-disorder transition and a stable Fddd region.

Near the same time in 2001, Reiter et al. [37] proposed a variant of FTS that avoids this issue by instead simulating $H_{f}\left[W_{-},w_{+}\right]$, where $W_{+}\left(r\right)$ is replaced by the saddle point $w_{+}\left(r\right)$. The advantage of this partial saddle-point approximation is that $w_{+}\left(r\right)$ is real valued, permitting the use of conventional simulation techniques. The justification for the approximation is that the fluctuations in $W_{-}\left(r\right)$ are far more important than those in $W_{+}\left(r\right)$, and indeed all evidence so far indicates that the approximation is accurate. Shortly after its introduction, Duchs et al. [38,39] used this variant of FTS to study ternary blends of diblock copolymer with its two parent homopolymers, but then the method received no further attention until 2013 when it was revived by Stasiak and Matsen [40]. The subsequent development, however, has resulted in FTS algorithms capable of problems beyond the reach of traditional particle-based simulations. As such, it is certain to become an important tool in the theoretical study of block copolymer systems. Here, we provide a tutorial on the method followed by demonstrations of its capabilities. To encourage its use, we also provide open-source code for neat diblock copolymer melts that can be readily modified to handle more complex systems.

## 2. Field-Theoretic Simulations

This section develops the FTS method starting from the underlying particle-based model, where the Hamiltonian, $H_{p}\left[\left\{r_{\alpha ,i}\right\}\right]$, is a function of all the particle coordinates, $\left\{r_{\alpha ,i}\right\}$. The first step involves a transformation to the field-based model, where the Hamiltonian $H_{f}\left[W_{-},W_{+}\right]$ is specified in terms of the fields, $W_{-}\left(r\right)$ and $W_{+}\left(r\right)$. The evaluation of $H_{f}\left[W_{-},W_{+}\right]$ requires the statistical mechanics of noninteracting polymers in the fields, which represents one of the two highly computational parts of the simulation. We explain how this is done numerically using fast Fourier transforms. Next, the pressure field, $W_{+}\left(r\right)$, is approximated by its saddle point, $w_{+}\left(r\right)$, which is the other highly computational part. We describe how to locate $w_{+}\left(r\right)$ iteratively using Anderson mixing [41]. The composition field, $W_{-}\left(r\right)$, is then evolved using conventional Langevin dynamics. The section concludes by discussing the Morse calibration, which converts the bare $\chi_{b}$ parameter used in the simulations to an effective χ parameter corresponding to the standard GCM.

### 2.1. Particle-Based Model

Most FTS have been based on continuous Gaussian chains. However, this requires numerical integration along the chain contours, which then leads to numerical inaccuracies. To avoid this, we will instead model the *n* polymers of the system by discrete bead-spring chains each with *N* monomers [42]. The position of monomer *i* of molecule α will be denoted by the vector $r_{\alpha ,i}$. In this particle-based representation, the Hamiltonian

(1) $$H_{p}\left[\left\{r_{\alpha ,i}\right\}\right]=U_{b}\left[\left\{r_{\alpha ,i}\right\}\right]+U_{\mathrm{int}}\left[\left\{r_{\alpha ,i}\right\}\right]$$

is divided into bonded and nonbonded interactions each expressed in terms of the particle coordinates. The bonded interactions take the simple form

(2) $$\frac{U_{b}\left[\left\{r_{\alpha ,i}\right\}\right]}{k_{B}T}=\frac{3}{2a^{2}}\sum_{\alpha =1}^{n}\sum_{i=1}^{N-1}{\left|r_{\alpha ,i}-r_{\alpha ,i+1}\right|}^{2}$$

which treats them as harmonic springs. Here, the spring constant is expressed in terms of the natural bond length, *a*. For simplicity, we assume conformational symmetry, but it is straightforward to assign different statistical lengths, $a_{A}$ and $a_{B}$, to the A-A and B-B bonds, respectively. The nonbonded interactions are restricted to contact forces between the A and B monomers. As such, their energy can be expressed as

(3) $$\frac{U_{\mathrm{int}}\left[\left\{r_{\alpha ,i}\right\}\right]}{k_{B}T}=\rho_{0}\chi_{b}\int {\hat{\phi }}_{A}\left(r\right){\hat{\phi }}_{B}\left(r\right)dr$$

where $\chi_{b}$ is the bare interaction parameter and

(4) $$\begin{array}{ccc} {\hat{\phi }}_{A}\left(r\right) & = & \frac{1}{\rho_{0}}\sum_{\alpha =1}^{n}\sum_{i=1}^{N_{A}}\delta \left(r-r_{\alpha ,i}\right) \end{array}$$

(5) $$\begin{array}{ccc} {\hat{\phi }}_{B}\left(r\right) & = & \frac{1}{\rho_{0}}\sum_{\alpha =1}^{n}\sum_{i=1}^{N_{B}}\delta \left(r-r_{\alpha ,i+N_{A}}\right) \end{array}$$

are dimensionless concentrations of the A and B monomers, respectively. It will become convenient to reexpress the A and B concentrations in terms of the composition, ${\hat{\phi }}_{-}\left(r\right)$, and total concentration, ${\hat{\phi }}_{+}\left(r\right)$, defined by

(6) $${\hat{\phi }}_{\pm }\left(r\right)\equiv {\hat{\phi }}_{A}\left(r\right)\pm {\hat{\phi }}_{B}\left(r\right)$$

In terms of these new concentrations, the energy of the nonbonded interactions becomes

(7) $$\frac{U_{\mathrm{int}}\left[\left\{r_{\alpha ,i}\right\}\right]}{k_{B}T}=\frac{\rho_{0}\chi_{b}}{4}\int \left[{\hat{\phi }}_{+}^{2}\left(r\right)-{\hat{\phi }}_{-}^{2}\left(r\right)\right]dr$$

As a result of incompressibility (i.e., ${\hat{\phi }}_{+}\left(r\right)=1$), the first term reduces to a constant, $\rho_{0}V\chi_{b}/4$, and therefore is typically dropped. However, we retain it because of its dependence on $\chi_{b}$, which will have implications regarding an ultraviolet divergence discussed later [42].

To determine the equilibrium behavior of this model, we need to perform statistical mechanics. This requires calculation of the partition function

(8) $$Z\propto \int \exp\left(-\frac{H_{p}\left[\left\{r_{\alpha ,i}\right\}\right]}{k_{B}T}\right)\delta \left[{\hat{\phi }}_{+}-1\right]d\left\{r_{\alpha ,i}\right\}$$

Note that the simple particle-based Hamiltonian in Equation (1) lacks any energy penalty for deviations from melt density, and therefore a Dirac delta functional has to be inserted to enforce incompressibility. Although the form of the partition function remains relatively simple, the integrations over the 3nN particle coordinates are nevertheless impossible to perform, which is the motivation for switching from particle coordinates to fields.

### 2.2. Field-Based Model

The transformation [14,15,16,18] to a field-based representation employs a Hubbard–Stratonovich identity for the Boltzmann weight

(9) $$\exp\left(-\frac{U_{\mathrm{int}}\left[\left\{r_{\alpha ,i}\right\}\right]}{k_{B}T}\right)\propto \int \exp\left(-\rho_{0}\int \left[\frac{\chi_{b}}{4}+\frac{W_{-}^{2}}{\chi_{b}}+W_{-}{\hat{\phi }}_{-}\right]dr\right)DW_{-}$$

involving a functional integral over a composition field, $W_{-}\left(r\right)$, and a Fourier representation of the Dirac delta functional

(10) $$\delta \left[{\hat{\phi }}_{+}-1\right]\propto \int \exp\left(-\rho_{0}\int W_{+}\left[{\hat{\phi }}_{+}-1\right]dr\right)DW_{+}$$

involving a functional integral over a pressure field, $W_{+}\left(r\right)$. Once the identities are inserted into Equation (8), the 3nN integrations over the particle coordinates can be performed analytically leaving just the two integrations over $W_{\pm }\left(r\right)$. The partition function can then be recast as

(11) $$Z\propto \int \exp\left(-\frac{H_{f}\left[W_{-},W_{+}\right]}{k_{B}T}\right)DW_{-}DW_{+}$$

where the field-based Hamiltonian takes the form

(12) $$\frac{H_{f}\left[W_{-},W_{+}\right]}{k_{B}T}=-n\ln Q\left[W_{-},W_{+}\right]+\rho_{0}\int \left(\frac{\chi_{b}}{4}+\frac{W_{-}^{2}}{\chi_{b}}-W_{+}\right)dr$$

involving a relatively simple single-chain partition function

(13) $$Q\left[W_{-},W_{+}\right]\propto \int \exp\left(-\frac{H_{1}\left[\left\{r_{i}\right\}\right]}{k_{B}T}\right)d\left\{r_{i}\right\}$$

for an individual molecule in an equivalent system of noninteracting polymers acted upon by only the fields. The Hamiltonian for $Q[W_{-},W_{+}]$ is given by

(14) $$\frac{H_{1}\left[\left\{r_{i}\right\}\right]}{k_{B}T}=\frac{U_{b}\left[\left\{r_{i}\right\}\right]}{k_{B}T}+\int \left[W_{-}{\hat{\phi }}_{-}+W_{+}{\hat{\phi }}_{+}\right]dr$$

where the bond energy and concentrations are for a single molecule (i.e., n=1), which allows us to drop the α index on the particle coordinates, $r_{i}$.

### 2.3. System of Noninteracting Polymers

The single-chain partition function can be conveniently reexpressed as

(15) $$Q=\frac{1}{V}\int \prod_{i=1}^{N-1}g(|r_{i+1}-r_{i}\left|\right)\prod_{i=1}^{N}h_{i}\left(r_{i}\right)\prod_{i=1}^{N}dr_{i}$$

where the Boltzmann weight in the integrand of Equation (13) is written as a product of separate factors for the N−1 bonds and the *N* monomers. Here,

(16) $$g\left(R\right)={\left(\frac{3}{2\pi a^{2}}\right)}^{3/2}\exp\left(-\frac{3R^{2}}{2a^{2}}\right)$$

is the Boltzmann weight for an individual bond and

(17) $$h_{i}\left(r\right)=\exp\left(-W_{+}\left(r\right)-\gamma_{i}W_{-}\left(r\right)\right)$$

is the Boltzmann weight for the field acting on monomer *i*. To distinguish the two components of the copolymer, we define $\gamma_{i}=1$ for A monomers (i.e., $i\leq N_{A}$) and $\gamma_{i}=-1$ for B monomers (i.e., $i>N_{A}$).

To evaluate Equation (15), we define a partial partition function, $q_{i}\left(r\right)$, for the first *i* monomers of the chain with its *i*’th monomer constrained to position r. It is obtained recursively using

(18) $$q_{i+1}\left(r\right)=h_{i+1}\left(r\right)\int g\left(R\right)q_{i}\left(r-R\right)dR$$

starting from $q_{1}\left(r\right)=h_{1}\left(r\right)$ [16,43]. Similarly, we define an analogous partial partition function, $q_{i}^{†}\left(r\right)$, for the last N+1−i monomers, which is obtained by iterating

(19) $$q_{i-1}^{†}\left(r\right)=h_{i-1}\left(r\right)\int g\left(R\right)q_{i}^{†}\left(r-R\right)dR$$

starting from $q_{N}^{†}\left(r\right)=h_{N}\left(r\right)$. Once both partial partition functions have been calculated, the single-chain partition function is given by

(20) $$Q=\frac{1}{V}\int \frac{q_{i}\left(r\right)q_{i}^{†}\left(r\right)}{h_{i}\left(r\right)}dr$$

The integral for *Q* can be evaluated using any value of 1≤i≤N, but one typically sets i=N. We will also require ensemble averages of the composition and total concentration in the system of noninteracting polymers, which are given by

(21) $$\begin{array}{ccc} \phi_{-}\left(r\right) & = & \frac{1}{NQ}\sum_{i=1}^{N}\gamma_{i}\frac{q_{i}\left(r\right)q_{i}^{†}\left(r\right)}{h_{i}\left(r\right)} \end{array}$$

(22) $$\begin{array}{ccc} \phi_{+}\left(r\right) & = & \frac{1}{NQ}\sum_{i=1}^{N}\frac{q_{i}\left(r\right)q_{i}^{†}\left(r\right)}{h_{i}\left(r\right)} \end{array}$$

respectively.

### 2.4. Numerical Method

The recursion relation in Equation (18) is calculated using the convolution theorem. This is done by taking a Fourier transform of the propagator

(23) $$q_{i}\left(k\right)=F\left[q_{i}\left(r\right)\right]\equiv \int q_{i}\left(r\right)\exp\left(-ik\cdot r\right)dr$$

and multiplying by

(24) $$g\left(k\right)=F\left[g(R)\right]=\exp\left(-\frac{a^{2}k^{2}}{6}\right)$$

The inverse Fourier transform of the product provides the integral

(25) $$\int g\left(R\right)q_{i}\left(r-R\right)dR=F^{-1}\left[g\left(k\right)q_{i}\left(k\right)\right]$$

which is then multiplied by $h_{i+1}\left(r\right)$ to give $q_{i+1}\left(r\right)$. The second recursion relation in Equation (19) is calculated in an analogous manner.

The above equations are generally solved in an orthorhombic $L_{x}\times L_{y}\times L_{z}$ simulation box of volume $V=L_{x}L_{y}L_{z}$ with periodic boundary conditions. The box is overlaid with an $m_{x}\times m_{y}\times m_{z}$ grid of uniform spacing (i.e., $\Delta_{\alpha }=L_{\alpha }/m_{\alpha }$ for α=x, *y*, and *z*). As such, each grid point corresponds to a volume of $V_{\mathrm{cell}}=\Delta_{x}\Delta_{y}\Delta_{z}$. Spatially-dependent quantities are then converted into arrays over the $M=m_{x}m_{y}m_{z}$ vertices of the grid. Likewise, we define an analogous grid in Fourier space that extends from $-\pi /\Delta_{\alpha }$ to $\pi /\Delta_{\alpha }$ with a spacing of $2\pi /L_{\alpha }$ in each direction α. The Fourier-space grid also contains *M* vertices, but approximately half of them are redundant for real-valued functions, f(r), due to the fact that f(−k) equals the complex conjugate of f(k).

### 2.5. Partial Saddle-Point Approximation

Although the two forms of the partition function in Equations (8) and (11) are mathematically equivalent, the Boltzmann weight in the field-based representation cannot be interpreted in terms of probability thus precluding the use of conventional simulation techniques. This is because the integration of the pressure field is performed along the imaginary axis from $W_{+}\left(r\right)=-i\infty$ to +i∞, which makes the Boltzmann weight complex valued. However, this problem can be overcome by evaluating the integral over $W_{+}\left(r\right)$ using the saddle-point approximation. Given that the Boltzmann weight is an analytic function of $W_{+}\left(r\right)$, the integration path can be deformed in the complex plane so as to pass through the saddle point of the Hamiltonian along a trajectory of constant phase, which concentrates the integration to the saddle-point region. Ignoring irrelevant prefactors, the partition function reduces to

(26) $$Z\propto \int \exp\left(-\frac{H_{f}\left[W_{-},w_{+}\right]}{k_{B}T}\right)DW_{-}$$

where $w_{+}\left(r\right)$ denotes the value of $W_{+}\left(r\right)$ at the saddle point. As such, $w_{+}\left(r\right)$ is determined by solving $DH_{f}/DW_{+}=0$, which equates to the mean-field incompressibility condition

(27) $$\phi_{+}\left(r\right)=1$$

Not only does the partial saddle-point approximation reduce *Z* to a single functional integration, $w_{+}\left(r\right)$ is real valued and thus the statistical mechanics can then be performed using standard techniques. The approximation does, however, require $w_{+}\left(r\right)$ to be reevaluated every time $W_{-}\left(r\right)$ changes.

### 2.6. Anderson Mixing

The saddle point, $w_{+}\left(r\right)$, is updated iteratively using the Anderson-mixing scheme [41,44,45,46]. To facilitate this, we label the saddle-point values by $w_{+,j}^{\left(k\right)}$, where j=1,2,…,M is the grid index and k=1,2,3,… is the iteration index. The iterations begin anew from k=1 after each change in the composition field, $W_{-}\left(r\right)$, generally starting with $w_{+,j}^{\left(1\right)}$ equal to the saddle-point solution corresponding to the preceding composition field.

The first step of the *k*’th iteration is to evaluate the deviation from incompressibility

(28) $$d_{j}^{\left(k\right)}=\phi_{+,j}^{\left(k\right)}-1$$

at each grid point, *j*. The overall error is then quantified by

(29) $$\varepsilon \equiv {\left[\frac{1}{M}\sum_{j=1}^{M}{\left(d_{j}^{\left(k\right)}\right)}^{2}\right]}^{1/2}$$

If the error exceeds some given tolerance, then an improved estimate, $w_{+,j}^{\left(k+1\right)}$, is obtained from the preceding $n_{r}$ iterations. This is done by evaluating the symmetric matrix

(30) $$U_{mn}=\sum_{j=1}^{M}\left(d_{j}^{\left(k\right)}-d_{j}^{\left(k-m\right)}\right)\left(d_{j}^{\left(k\right)}-d_{j}^{\left(k-n\right)}\right)$$

and vector

(31) $$V_{m}=\sum_{j=1}^{M}\left(d_{j}^{\left(k\right)}-d_{\gamma ,j}^{\left(k-m\right)}\right)d_{j}^{\left(k\right)}$$

where $m,n=1,2,\ldots ,n_{r}$. From these, we calculate coefficients

(32) $$C_{n}=\sum_{m=1}^{n_{r}}{\left(U^{-1}\right)}_{nm}V_{m}$$

used to combine the fields and deviations from past histories as

(33) $$\begin{array}{ccc} W_{+,j}^{\left(k\right)} & = & w_{+,j}^{\left(k\right)}+\sum_{n=1}^{n_{r}}C_{n}\left(w_{+,j}^{\left(k-n\right)}-w_{+,j}^{\left(k\right)}\right) \end{array}$$

(34) $$\begin{array}{ccc} D_{j}^{\left(k\right)} & = & d_{j}^{\left(k\right)}+\sum_{n=1}^{n_{r}}C_{n}\left(d_{j}^{\left(k-n\right)}-d_{j}^{\left(k\right)}\right) \end{array}$$

respectively. The next iteration of the field is then obtained by mixing the fields and deviations together as follows

(35) $$w_{+,j}^{\left(k+1\right)}=W_{+,j}^{\left(k\right)}+\lambda D_{\gamma ,j}^{\left(k\right)}$$

where λ>0 is referred to as the mixing parameter. In the absence of any histories (i.e., $n_{r}=0$), Anderson mixing reduces to what is generally referred to as simple mixing.

The convergence tends to be faster for larger λ, but it becomes unstable if λ is too large. Increasing the number of histories improves the convergence as well as the stability, but only up to a point. Therefore, based on previous experience [45], we increase the number of histories up to a maximum of $n_{h}=20$ while ramping up the mixing parameter to a maximum of *N*. This is done by setting $n_{r}=\min\left(k-1,n_{h}\right)$ and $\lambda =N(1.0-0.9^{k})$. We typically continue the iterations until $\varepsilon \leq 10^{-4}$.

### 2.7. Langevin Dynamics

A Markov sequence of configurations for the partition function in Equation (26) is generated by evolving the composition field, $W_{-}\left(r;\tau \right)$, using Langevin dynamics,

(36) $$W_{-}\left(r;\tau +\delta \tau \right)=W_{-}\left(r;\tau \right)-\Lambda_{\ell }\delta \tau +N\left(0,\sigma \right)$$

where thermal noise is provided by random numbers, N(0,σ), generated from a normal distribution of zero mean and $\sigma^{2}=2\delta \tau /V_{\mathrm{cell}}\rho_{0}$ variance. To improve accuracy, we apply the predictor-corrector algorithm [47,48]. A predicted field at τ+δτ is first obtained using Equation (36) with

(37) $$\Lambda_{1}=\phi_{-}\left(r;\tau \right)+\frac{2}{\chi_{b}}W_{-}\left(r;\tau \right)$$

and then a corrected field is obtained with

(38) $$\Lambda_{2}=\frac{1}{2}\left[\Lambda_{1}+\phi_{-}\left(r;\tau +\delta \tau \right)+\frac{2}{\chi_{b}}W_{-}\left(r;\tau +\delta \tau \right)\right]$$

evaluated using the predicted field and corresponding composition. Note that the predictor and corrector steps use the same set of random numbers.

As usual, the Langevin dynamics are applied for a sufficient amount of time, $\tau_{\mathrm{eq}}$, to equilibrate the system before observables are sampled. There is generally significant correlation between successive time steps and so it is common to only sample periodically, such as once every 10δτ. Naturally, one is interested in the physical quantities of the particle-based model. It is therefore necessary to relate these quantities to those of the field-based model. The average composition, for example, is given by [37]

(39) $$\langle {\hat{\phi }}_{-}\left(r\right)\rangle =-\frac{2}{\chi_{b}}\langle W_{-}\left(r\right)\rangle$$

and the two-point correlation function is given by [37]

(40) $$\langle {\hat{\phi }}_{-}\left(r\right){\hat{\phi }}_{-}\left(r^{\prime }\right)\rangle =\frac{4}{\chi_{b}^{2}}\langle W_{-}\left(r\right)W_{-}\left(r^{\prime }\right)\rangle -\frac{2N^{2}\delta \left(r-r^{\prime }\right)}{\chi_{b}\rho_{0}}$$

It can be advantageous to evaluate the average composition using $\langle {\hat{\phi }}_{-}\left(r\right)\rangle =\langle \phi_{-}\left(r\right)\rangle$, because the composition in the system of noninteracting polymers fluctuates less than the composition field. However, be aware that $\langle {\hat{\phi }}_{-}\left(r\right){\hat{\phi }}_{-}\left(r^{\prime }\right)\rangle \neq \langle \phi_{-}\left(r\right)\phi_{-}\left(r^{\prime }\right)\rangle$. Another quantity of interest is the structure function,

(41) $$\frac{S(k)}{\rho_{0}N}=\frac{n}{{\left(\chi_{b}V\right)}^{2}}\langle |W_{-}\left(k\right){|}^{2}\rangle -\frac{1}{2\chi_{b}N}$$

which is proportional to the Fourier transform of the two-point correlation function in Equation (40).

### 2.8. Effective Interaction Parameter

Because we switched from continuous to discrete chains, our results need to be mapped back onto the standard GCM. Furthermore, there is an ultraviolet (UV) divergence that occurs as $\Delta_{\alpha }\to 0$, which generally requires renormalization of χ and *a* [29,49]. In this case, however, the divergence in the segment length is avoided by our use of the partial saddle-point approximation. As it turns out, the change in the model and the UV divergence can be dealt with simultaneously by employing the Morse calibration [4,6].

For large *N*, the interactions are generally weak and therefore the dependence of the effective χ on the bare $\chi_{b}$ can be approximated by the linear relationship [6]

(42) $$\chi \approx z_{\infty }\chi_{b}$$

where the proportionality factor, $z_{\infty }$, is given by the relative number of intermolecular contacts in the limit of $\chi_{b}\to 0$ and N→∞. This factor is conveniently expressed as

(43) $$z_{\infty }=1-\frac{1}{V_{\mathrm{cell}}\rho_{0}}\sum_{i=-\infty }^{\infty }P_{i}$$

where $V_{\mathrm{cell}}\rho_{0}$ is the total number of contacts experienced by a given monomer and $P_{i}$ is the probability that two monomers of an infinite chain, separated by *i* bonds along the contour, occupy the same cell of the grid. For orthorhombic simulation boxes [42],

(44) $$P_{i}=\prod_{\alpha }\frac{\Delta_{\alpha }}{a}\sqrt{\frac{3}{2\pi |i|}}\;erf\left(\frac{\pi a}{\Delta_{\alpha }}\sqrt{\frac{\left|i\right|}{6}}\right)$$

Note that $P_{0}=1$. In the case of cubic grids with a resolution less than the bond length (i.e., Δ≳a), Equation (43) reduces to

(45) $$z_{\infty }\approx 1-\frac{2.3327R_{0}}{\sqrt{\bar{N}}\Delta }$$

which is the proportionality factor first used by Stasiak and Matsen [40].

**Figure 1:**
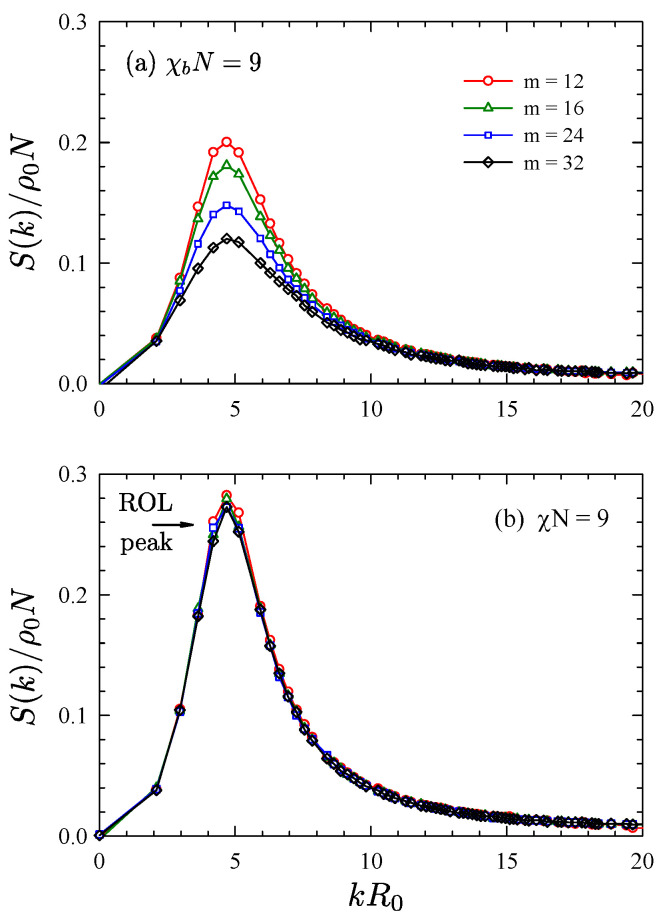
Structure function, S(k), for symmetric diblocks of $\bar{N}=10^{4}$ obtained in a cubic simulation box of size $L=3R_{0}$ with different numbers of grid points, m=L/Δ. Plot (**a**) shows results for $\chi_{b}N=9$ without any calibration, while plot (**b**) shows results for χN=9 using the linear calibration, $\chi =z_{\infty }\chi_{b}$. The arrow marks the peak height predicted by ROL theory. Reproduced from Ref. [42].

Figure 1 illustrates the quality of the linear calibration by comparing the structure function, S(k), at different grid resolutions for $\bar{N}=10^{4}$. Plot (**a**) demonstrates what happens if the UV divergence is ignored. When $\chi_{b}N$ is held constant, the segregation decreases as Δ=L/m→0, which is evident by a reduction in the peak height of S(k). Plot (**b**) corrects for this by comparing S(q) at a fixed $z_{\infty }\chi_{b}N$, which results in good agreement over the full range of resolutions. Furthermore, the resulting peak height is similar to that of the ROL prediction, which implies that the linear approximation, $\chi \approx z_{\infty }\chi_{b}$, is reasonably accurate for $\bar{N}=10^{4}$. However, the peak is slightly higher than the ROL prediction, which means that the true value of χ is somewhat larger.

**Figure 2:**
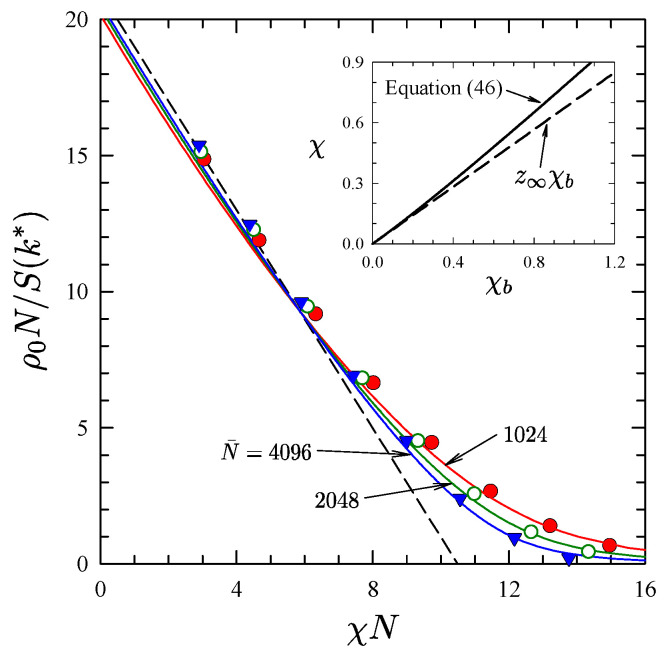
Inverse peak height of the structure function, $S^{-1}\left(k^{*}\right)$, plotted in terms of effective χ for different invariant polymerization indices, $\bar{N}$. Symbols denote FTS results, solid curves show the ROL predictions, and the dashed line is the RPA prediction. The linear and nonlinear χ in Equations (42) and (46) are plotted in the inset with dashed and solid lines, respectively. Reproduced from Ref. [48].

For smaller $\bar{N}$, it becomes necessary to go beyond the linear approximation of χ. In the Morse calibration, the nonlinear correction is calculated by fitting the peak of the structure function, $S(k^{*})$, from FTS to ROL predictions for symmetric diblocks. Beardsley and Matsen [13,48] demonstrated the calibration for Δ=a and $\rho_{0}=8/a^{3}$, assuming the empirical relationship [4]

(46) $$\chi =\frac{z_{\infty }\chi_{b}+c_{1}\chi_{b}^{2}}{1+c_{2}\chi_{b}}$$

between the bare $\chi_{b}$ of the FTS and the effective χ of the ROL theory. The resulting calibration, $z_{\infty }=0.7084$, $c_{1}=1.246$, and $c_{2}=1.367$, is plotted in the inset of Figure 2. The main plot of Figure 2 illustrates that the quality of the fit is generally good. There is, however, some significant deviation at weak segregations, where the FTS results coincide with the mean-field or rather random-phase approximation (RPA) [22] as opposed to the more accurate ROL theory. This is a consequence of the partial saddle-point approximation, and so it could potentially be remedied by performing FTS with fluctuations in both fields. Nevertheless, the inaccuracy remains acceptable for $\bar{N}≳10^{3}$.

## 3. Applications

The initial applications of FTS have largely focused on two test cases. The first one is neat diblock copolymer melts, which is a natural choice given its relative simplicity and the fact that it is the most thoroughly understood [13,33,34,40,42,48,50]. As mentioned, the greatest impact of fluctuations is on the disordered phase, and thus particular attention is paid to the order-disorder transition (ODT). The second one is ternary blends of diblock copolymer with its two parent homopolymers [38,39,51,52,53]. Naturally, this system has a far larger parameter space, and so studies have concentrated on symmetric blends where the diblock has a composition of f=0.5, the two homopolymers have equal polymerizations $N_{h}$, and the homopolymers have either equal concentrations or equal chemical potentials. For experimental convenience [54,55,56,57,58,59,60,61,62,63,64], the diblock polymerization is generally set to $N_{c}=N_{h}/\alpha$ with α=0.2, such that the ODT occurs at $\chi N_{h}\approx 2$ over the full range of diblock volume fractions, ${\bar{\phi }}_{c}$. In this system, particular attention is paid to a bicontinuous microemulsion (BμE), where the two homopolymers segregate into interweaving microdomains separated by a monolayer of diblock copolymer. The BμE is regarded as a pivotal test of the FTS, as it is a fluctuation-induced phase that is entirely absent from the SCFT phase diagram [38,39,52,65].

### 3.1. Diblock Copolymer Melts

The classical example of a fluctuation effect is the shift in the ODT of symmetric diblock copolymers from the SCFT prediction, ${\left(\chi N\right)}_{\mathrm{ODT}}=10.495$. The earliest treatment by Fredrickson and Helfand [24] predicted a shift of $41.0{\bar{N}}^{-1/3}$, where $\bar{N}\equiv a^{6}\rho_{0}^{2}N$ is the invariant polymerization index. Particle-based simulations of different models [8,9,10,11] have since provided the more accurate prediction

(47) $${\left(\chi N\right)}_{\mathrm{ODT}}=10.495+41.0{\bar{N}}^{-1/3}+123.0{\bar{N}}^{-0.56}$$

The ODT is generally found by matching the free energies of the ordered and disordered phases. Although FTS do not provide direct access to the free energy, ensemble averaging a derivative of the Hamiltonian provides the corresponding derivative of the free energy. These can then be integrated to obtain changes in free energy, so long as a phase transition does not occur along the integration path. Lennon et al. [33] were the first to apply thermodynamic integration to FTS. They integrated a composite Hamiltonian, $H=\lambda H_{f}+\left(1-\lambda \right)H_{\mathrm{ec}}$, from λ=0 to 1 in order to bridge between the known free energy of an Einstein crystal and the free energy of the polymeric system for a specified value of χ. One integration was performed to evaluate the free energy of the disordered phase below the transition and another to evaluate the free energy of the ordered phase above the transition. The ODT was then located by integrating with respect to the χ parameter.

**Figure 3:**
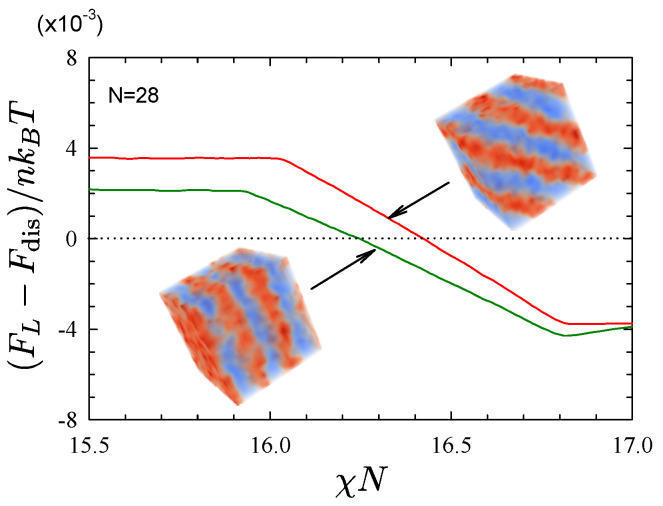
Free energy difference between lamellar and disordered phases, $F_{L}-F_{\mathrm{dis}}$, from thermodynamic integration for molecules of polymerization N=28 for two different lamellar periods. The kinks at small and large χN result when the metastable phase switches to the stable phase. Adapted from Ref. [48].

Beardsley and Matsen [48] located the ODT of symmetric diblocks using a slight variation of the technique where the ensemble average and integration are performed simultaneously [66]. Figure 3 shows the resulting free energy difference between the lamellar and disordered phases, $F_{L}-F_{\mathrm{dis}}$, for diblocks of N=28 and $\rho_{0}a^{3}=8$ in a cubic simulation box of size L=24a. The free energy comparison is performed for lamellar periods of D=8a and $D=24a/\sqrt{8}\approx 8.5a$, which are the periods that spontaneously form when the disordered phase is quenched into the ordered region. In this case, the thermodynamic integration implies that the shorter period is favored, given that it results in a lower ODT of ${\left(\chi N\right)}_{\mathrm{ODT}}=16.25$.

**Figure 4:**
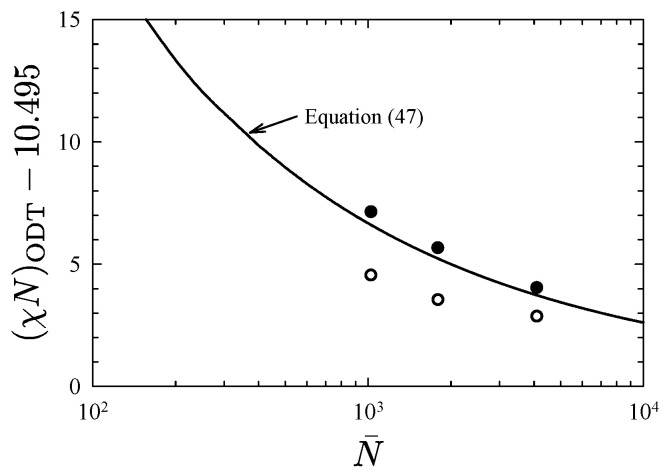
Fluctuation correction to the ODT of symmetric diblock copolymers. The open and closed symbols are based on the linear, $\chi =z_{\infty }\chi_{b}$, and nonlinear, Equation (46), interaction parameters, respectively. The curve compares the universal prediction from Equation (47) [8]. Adapted from Ref. [48].

Figure 4 compares the ODTs of different sized diblocks to Equation (47). The open and closed symbols denote their positions based on the linear and nonlinear interaction parameters in Equations (42) and (46), respectively. The linear χ results in considerable inaccuracy for $\bar{N}≲10^{4}$, but this is largely corrected for by the nonlinear χ. Still, there is a slight deviation for the shorter polymers, which could be due to inaccuracy in the partial saddle-point approximation. It could also be due to a breakdown in universality, which will ultimately occur if *N* becomes too small.

A more recent study [42] mapped the ODTs over a range of compositions, *f*, for diblock copolymers of a fixed N=90. In that study, the FTS were performed at a sufficiently large $\bar{N}=10^{4}$ to justify the use of the linear χ. However, at this relatively large $\bar{N}$, the thermodynamic integration became problematic for the nonlamellar phases. The relative ease of forming defects created noisy free energy curves and the resulting lack of metastability prevented a clear crossing of the curves. Nevertheless, because of the lack of metastability, the ODT could instead be located by monitoring the disappearance of Bragg peaks in the structure function of the ordered phase, S(k), as χN was slowly decreased.

**Figure 5:**
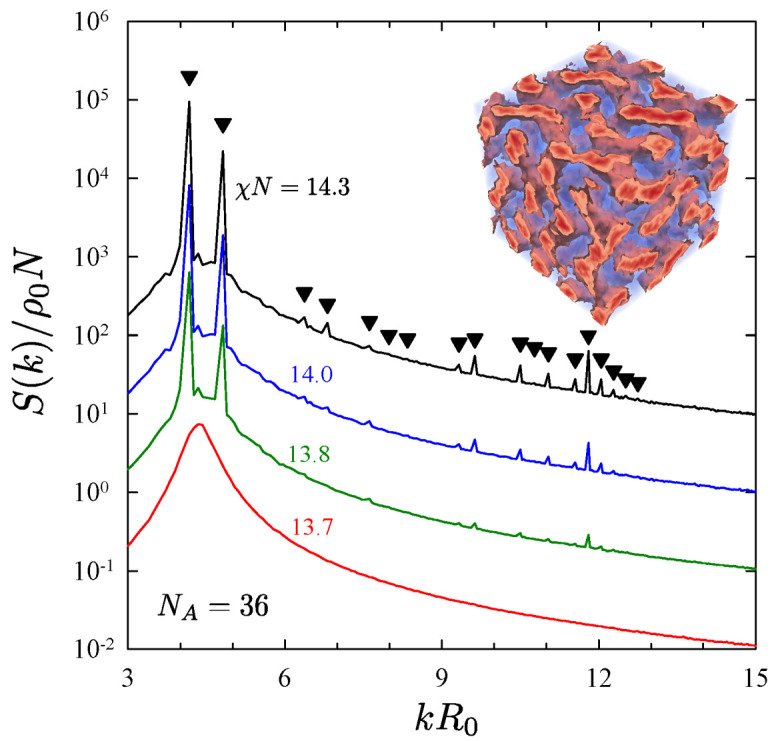
Structure function, S(k), at a sequence of χN values, calculated for diblocks with $N_{A}=36$ and $N_{B}=54$. For clarity, the curves for the ordered states exhibiting peaks are shifted up by an integer number of decades. The morphology (see inset) and peak positions (triangles) confirm that the ordered state is gyroid. Reproduced from Ref. [42].

Figure 5 illustrates the procedure for diblocks of $N_{A}=36$ and $N_{B}=54$. A single unit cell of the gyroid phase [67,68,69] was first obtained in a series of different-sized simulation boxes by quenching the disordered phase. The resulting morphology was then periodically repeated creating eight unit cells, and S(k) was evaluated revealing Bragg peaks consistent with the Ia3d symmetry of the gyroid phase. The box corresponding to the strongest peaks was assumed to be commensurate with the preferred period, and the segregation of that box was then reduced in small steps of 0.1 until the peaks disappeared at χN=13.7. The simulations at χN=13.8 were then repeated for a prolonged interval to ensure that the gyroid phase was indeed stable at the higher segregation. The fact that it was implies an ODT of ${\left(\chi N\right)}_{\mathrm{ODT}}=13.75\pm 0.05$. Notably, these are the first results to show that even weak fluctuations at $\bar{N}=10^{4}$ are sufficient to produce a direct gyroid-disorder transition, which is consistent with experiments on PE-PEP diblock copolymers [23]. Although the experiments actually observed a transition to perforated lamellae [70], this is understood to be an intermediate state that eventually converts to gyroid [71,72,73].

**Figure 6:**
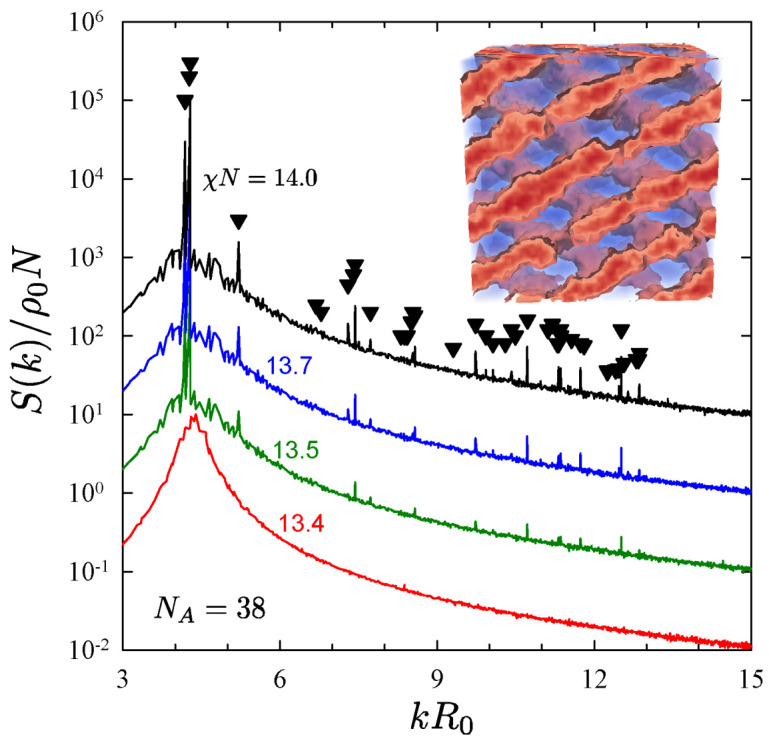
Analogous plot to that of Figure 5, but for diblocks with $N_{A}=38$ and $N_{B}=52$. The morphology (see inset) and peak positions (triangles) confirm that the ordered state is Fddd. Reproduced from Ref. [42].

Figure 6 repeats the procedure for $N_{A}=38$ and $N_{B}=52$, where disordered melts still show a clear preference for network structures. However, given the more symmetric composition, the Fddd phase [74] becomes a potential candidate. Indeed, while the gyroid phase disordered at χN=13.7, the Fddd phase remained stable down to χN=13.4 as shown in Figure 6. Furthermore, we have subsequently confirmed that when the segregation of the disordered melt at χN=13.4 is increased back to χN=13.5, the first three peaks in S(k) reappear. Although the defects are too long lived for the higher order peaks to return, it is clear that the Fddd phase is stable at χN=13.5, which then implies an ODT of ${\left(\chi N\right)}_{\mathrm{ODT}}=13.45\pm 0.05$. Previous calculations [26] and simulations [34] have suggested that fluctuations destroy the Fddd phase, contrary to its observation in PS-PI diblock copolymers melts [27,28]. These new FTS results are the first to corroborate the experiments.

**Figure 7:**
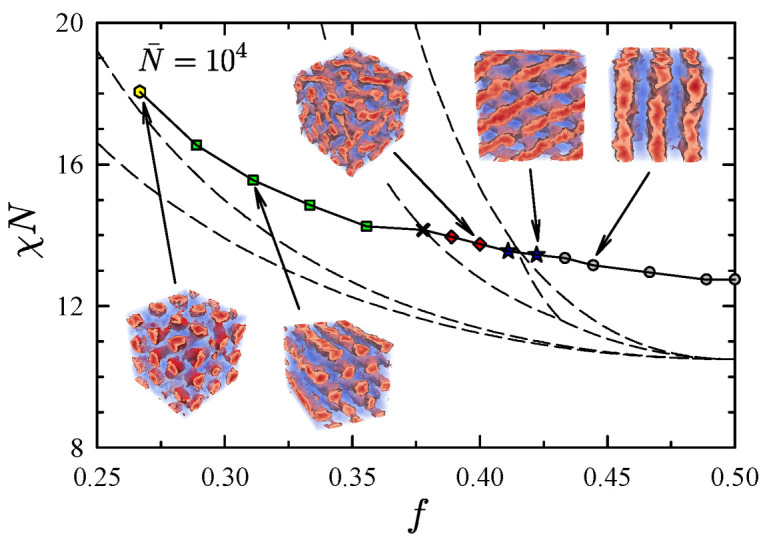
Order-disorder transitions for diblock copolymer melts at $\bar{N}=10^{4}$. The different symmetries of the ordered phases are denoted by circles (lamellar), stars (Fddd), diamonds (gyroid), squares (cylindrical), and hexagons (spherical). The cross marks an ODT for which the relative stability of the cylindrical and gyroid phases was indistinguishable. For comparison purposes, the SCFT phase diagram [21] is overlaid with dashed curves. Adapted from Ref. [42].

Figure 7 compares the above ODTs and others to the SCFT phase diagram. Consistent with experiments [23], the ODT is shifted upwards relative to the SCFT prediction producing direct transitions between the disordered phase and the ordered lamellar, Fddd, gyroid, cylindrical and spherical phases. The preferred symmetries of the ordered phases were obvious for most of the compositions. The only exception was at $N_{A}=34$ and $N_{B}=56$ (denoted by a cross in Figure 7), where the cylindrical and gyroid phases both remained stable down to χN=14.2. Nevertheless, this is consistent with the fact that this composition coincides closely with the cylindrical-gyroid boundary in the SCFT phase diagram.

Interestingly, the fluctuation-induced shift of the ODT in Figure 7 is reasonably uniform across the range of compositions. This, in fact, agrees with particle-based simulations, which observe shifts of 2.61 and 2.65 at f=0.25 and 0.5, respectively, [8,12]. However, the shift predicted by FTS is somewhat smaller by about 0.4. Based on the results in Figure 4, this is readily attributed to the inaccuracy of the linear $\chi =z_{\infty }\chi_{b}$.

### 3.2. Ternary Diblock–Homopolymer Blends

The addition of extra molecular species, A- and B-type homopolymers in this case, leads to the possibility of macrophase separation. To deal with this, it is convenient to work in the grand-canonical ensemble, where the concentrations of the different species are controlled by chemical potentials. This generally poses a problem for particle-based simulations because of the difficulty of inserting large macromolecules into a dense melt [75,76]. However, there is no such problem in FTS, and in fact only minor modifications are required to switch between different systems and ensembles [51]. The first term in the Hamiltonian of Equation (12) just needs to be substituted by the free energy of noninteracting molecules corresponding to the system of interest in the ensemble of interest. Similarly, the expressions for $\phi_{-}\left(r\right)$ and $\phi_{+}\left(r\right)$ in Equations (21) and (22) are replaced by the composition and total concentration for the same noninteracting system.

**Figure 8:**
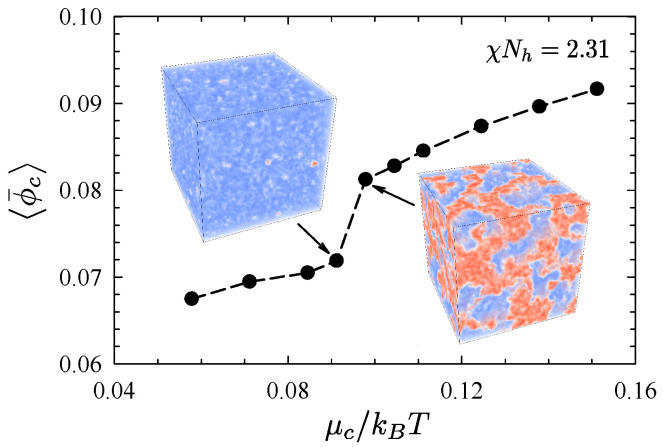
Average copolymer content in the ternary blends as a function of chemical potential for $\chi N_{h}=2.31$, obtained from grand-canonical FTS using cubic simulation boxes of size $72R_{h}$. Adapted from Ref. [53].

Figure 8 shows results from a grand-canonical simulation performed in a cubic simulation box of size $L=72R_{h}$ [53]. In particular, it shows the average copolymer concentration, $\langle {\bar{\phi }}_{c}\rangle$, as a function of the copolymer chemical potential, $\mu_{c}$, for a blend with ${\bar{N}}_{h}=10^{4}$, ${\bar{N}}_{c}=5\times 10^{4}$, and $\chi N_{h}=2.31$. Here, the linear $\chi =z_{\infty }\chi_{b}$ is used and lengths are expressed in terms of the unperturbed end-to-end length of a homopolymer molecule, $R_{h}=aN_{h}^{1/2}$. Interestingly, the plot exhibits a sudden jump in copolymer concentration near $\mu_{c}\approx 0.1k_{B}T$. Inspection of the configurations (see insets) reveals that this is due to a transition from a homopolymer rich phase at low $\mu_{c}$ to a bicontinuous microemulsion (BμE) at high $\mu_{c}$. As a result of the symmetry, the A-homopolymer and B-homopolymer rich phases are identical in free energy. Thus, the low-$\mu_{c}$ region corresponds to two-phase coexistence (A+B) and the transition corresponds to three-phase coexistence (A+B+BμE). Based on the jump in $\langle {\bar{\phi }}_{c}\rangle$, the binodals of the three-phase region are approximately ${\bar{\phi }}_{c}\approx 0.072$ and 0.082. However, there is some inaccuracy in these values due to the uncertainty regarding the chemical potential of coexistence.

**Figure 9:**
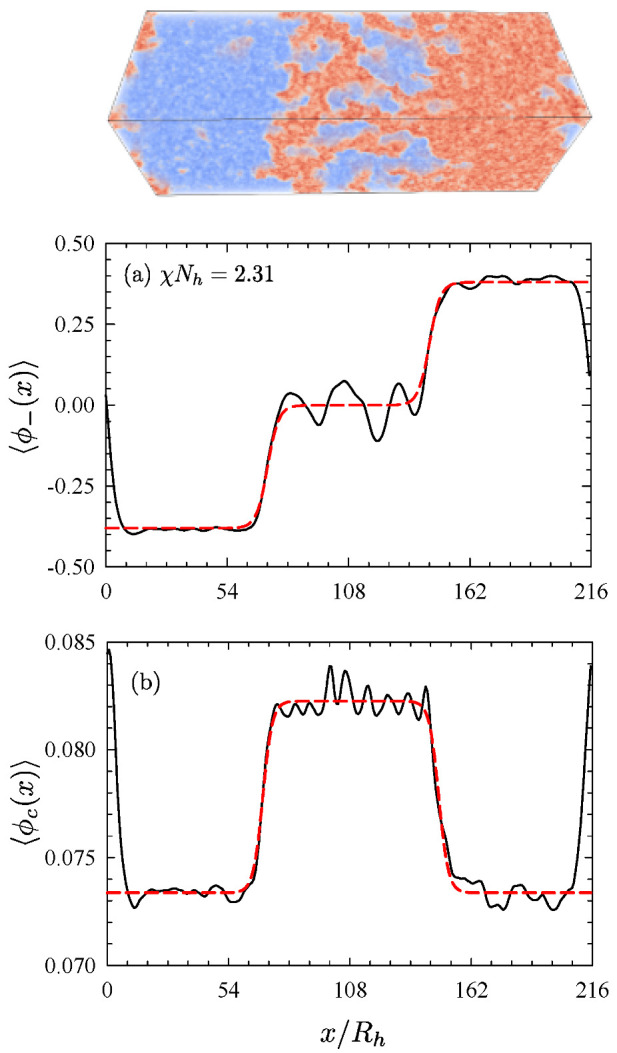
(**a**) Composition and (**b**) copolymer concentration profiles for three-phase coexistence at $\chi N_{h}=2.31$. The dashed curves are fits to hyperbolic tangent profiles. Adapted from Ref. [53].

With a sufficiently large simulation box, it is possible to simulate the three-phase coexistence in the canonical ensemble. Spencer and Matsen [53] did so by splicing together configurations of the three phases from the grand-canonical simulation (e.g., the insets of Figure 8), creating an orthorhombic simulation box of size $216R_{h}\times 72R_{h}\times 72R_{h}$. The image at the top of Figure 9 shows an equilibrated configuration with well-defined interfaces separating the three coexisting phases. Note that the simulation box used reflecting boundaries on all sides, and thus there is an A/B interface at x≈0 as well as two homopolymer/microemulsion interfaces at $x\approx 72R_{h}$ and $144R_{h}$. Figure 9a,b plot the composition and copolymer concentration averaged over the *y*-*z* plane as a function of the long dimension *x*. The dashed curves show fits using the standard hyperbolic profile for the interfaces [77]. The interfacial widths can be extracted directly from the fits and the interfacial tensions can be obtained from ensemble averages of appropriate derivatives of the Hamiltonian [51,53,78]. The fits also provide accurate binodals, ${\bar{\phi }}_{c}\approx 0.0734$ and 0.0823, due to the fact that the system is able to equilibrate the concentrations by adjusting the volumes of the coexisting phases.

**Figure 10:**
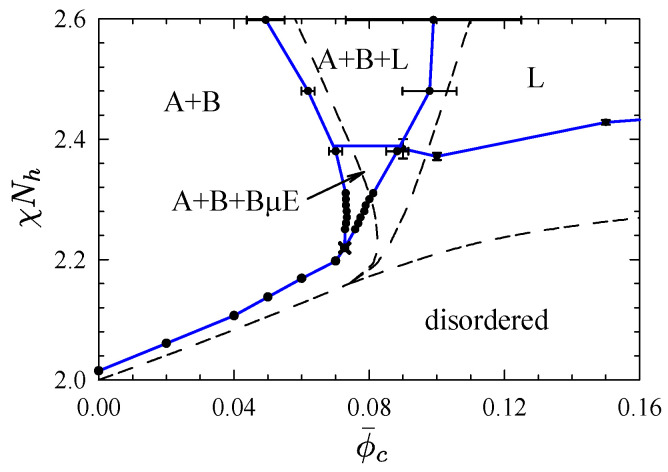
Phase diagram for symmetric ternary blends plotted in terms of segregation strength, $\chi N_{h}$, and copolymer content, ${\bar{\phi }}_{c}$. The labels A, B, L and BμE denote A-homopolymer rich, B-homopolymer rich, lamellar, and bicontinuous microemulsion, respectively. The cross marks the position of a tricritical point, and the dashed curves compare the SCFT phase diagram [38]. Adapted from Refs. [52,53].

The boundaries of the A+B+BμE region at $\chi N_{h}=2.31$ are included in the FTS phase diagram shown in Figure 10 [52,53]. As the diagram illustrates, the A+B+BμE coexistence switches to A+B+L coexistence for $\chi N_{h}≳2.38$ and it narrows in the opposite direction resulting in a tricritical point at $\chi N_{h}\approx 2.22$. Beyond that is a critical line separating A+B coexistence from disordered melts, referred to as the Scott line [79]. The Scott line was calculated using a finite-size scaling analysis [51,80,81], and the L/dis boundary was calculated in much the same way as it was for neat diblock copolymer melts. Note that the lamellar and disordered phases must, in principle, be separated by L+dis coexistence; however, the width of this coexistence was too narrow to resolve.

The SCFT phase diagram for this system [38], corresponding to ${\bar{N}}_{h}\to \infty$, is shown in Figure 10 with dashed curves. In this limit, the L/dis boundary and Scott line meet at a Lifshitz point, above which there is A+B+L coexistence. The effect of fluctuations is quite simple. As expected, fluctuations shift both the L/dis boundary and the Scott line to higher $\chi N_{h}$. However, the shift of the L/dis boundary is larger, and consequently it intersects the three-phase coexisting region splitting it into A+B+BμE below the point of intersection and A+B+L above.

Experiments [54,55,56,57,58,59,60,61,62,63,64] have proposed a somewhat different phase diagram, where the A+B and L regions are separated by a channel of BμE rather than three-phase coexistence. It is difficult to fathom how this proposed phase diagram could possibly converge to the SCFT diagram, which it must, in principle, do as ${\bar{N}}_{h}\to \infty$. It is entirely possible that the experiments overlooked the three-phase coexistence due to insufficient annealing. Indeed, a recent experiment by Xie et al. [82] has, in fact, observed macrophase separation. Thus, we believe that the topology of the FTS phase diagram in Figure 10 represents the true equilibrium behavior.

## 4. Discussion and Future Outlook

Even with relatively few applications, FTS have demonstrated remarkable potential. For instance, the simulations of three-phase coexistence in Figure 9 involve approximately $10^{8}$ molecules, which is orders of magnitude beyond what is possible in particle-based simulations [75]. This capability has been facilitated by a number of significant advances. For instance, the transition from CPUs to GPUs has allowed the system size to be scaled up considerably [83,84]. A similar improvement was also achieved by switching from Monte Carlo dynamics used in the early studies [13,38,39,40,50,51,80] to the Langevin dynamics in Equation (36) [48].

There will undoubtedly be further opportunities to improve the FTS method described here. One possibility is replacing the predictor-corrector algorithm with a better scheme [47]. There is also scope for tuning the Anderson-mixing algorithm, such as how the mixing parameter, λ, is adjusted. Alternatively, there may be a better numerical method for iterating $w_{+}\left(r\right)$ [85]. Naturally, the speed of the simulations would be enhanced by reducing the polymerization, *N*, although one must be careful not to go too far or otherwise universality will be lost. Likewise, it might be possible to increase the grid spacing, $\Delta_{\alpha }$, but it should nevertheless remain fine enough to resolve the relevant coarse-grained details such as the width of the internal A/B interfaces.

One of the main strengths of the field-theoretic approach is its incredible versatility. In particular, it is capable of handling complicated polymeric architectures with a minimal increase in computational effort relative to that of the simple diblock [86,87], which is most certainly not the case for traditional particle-based simulations. Furthermore, it can be adapted to a variety of ensembles [51,88,89], which is very useful when dealing with blends. For the case of AB-type systems, the only change involves the statistical mechanics for the noninteracting polymers. This just requires simple modifications to the first term in the field-theoretic Hamiltonian, Equation (12), and the expressions for $\phi_{\pm }\left(r\right)$, Equations (21) and (22). The extension to three or more chemically-distinct components (e.g., ABC-type systems) is also possible, although the modifications are less trivial [47].

It is important to remember that the version of FTS described here uses a partial saddle-point approximation, which equates to a mean-field enforcement of incompressibility. Past studies [38,90] have shown that the approximation is accurate, but it becomes less so as $\bar{N}$ is reduced. The clearest evidence for this is the inability to capture the departure of $S(k^{*})$ from the RPA prediction at small χN, as seen in Figure 2. Although complex-Langevin simulations could, in principle, capture this effect, it is uncertain whether, in practice, they can; the compressibility required to access the smaller values of $\bar{N}$ in Figure 2 may actually destroy the universality. In any case, the effect remains relatively minor for $\bar{N}≳10^{3}$, and thus the partial saddle-point approximation appears justified. For systems with more than two components, there will be additional fields that may also be imaginary, depending on the relative size of the different interaction parameters [47]. It is likely that they can also be treated accurately with the partial saddle-point approximation, although this remains to be seen.

Naturally, there will be some systems for which it is important to treat incompressibility rigorously, and thus the partial saddle-point approximation would be inappropriate or at least significantly inaccurate. An example is bottlebrush architectures involving backbones with densely-grafted side-chains. In these systems, steric interactions cause the side-chains to form a brush exerting a tension on the backbone, which reduces its flexibility. This steric stiffening of the backbone is completely neglected by mean-field treatments of incompressibility, but the effect does appear to be captured by complex-Langevin FTS [91]. Another example is block copolymer nanocomposites. Without a proper treatment, the nanoparticles can overlap and polymers can penetrate their interiors [92,93]. Although complex-Langevin FTS have been applied to these nanocomposites [35,88,94], they may not adequately account for the more significant steric interactions of bulky nanoparticles. Such steric interactions should remain in the limit of infinite $\bar{N}$, but this cannot possibly be the case in complex-Langevin FTS as they reduce to SCFT in that limit. On the other hand, complex-Langevin FTS do appear capable of handling the steric interactions in polymer solutions involving small solvent molecules [95].

Despite considerable progress, there remain some significant issues to resolve. An important one is determining the exact nature of the UV divergence, or in other words exactly how various quantities depend on the grid resolution. The calibration of the interaction parameter, χ, ensures that melts behave equivalently on different sized grids. Nevertheless, the free energy, for example, still retains a dependence on the grid resolution. This can be calculated analytically for homopolymer melts. In this special case, the field-based Hamiltonian reduces to

(48) $$\begin{array}{ccc} \frac{H_{f}\left[W_{-},w_{+}\right]}{k_{B}T} & = & \frac{\rho_{0}}{\chi_{b}}\int {\left(W_{-}\left(r\right)\pm \frac{\chi_{b}}{2}\right)}^{2}dr\\ & = & \frac{\rho_{0}V}{\chi_{b}M}\sum_{j=1}^{M}{\left(W_{-,j}\pm \frac{\chi_{b}}{2}\right)}^{2} \end{array}$$

where the plus and minus signs correspond to $N_{A}=N$ and $N_{A}=0$, respectively. In both cases, the Hamiltonian is just a system of *M* independent harmonic oscillators, and therefore its free energy is $\frac{1}{2}Mk_{B}T\ln\left(\chi_{b}M/\rho_{0}V\right)$ to within an irrelevant constant. The issue is that the free energy diverges as *M* increases or equivalently as $\Delta_{\alpha }\to 0$. Interestingly, we find that the UV divergence for diblock copolymer melts appears to be independent of composition (i.e., $N_{A}$) to within the numerical inaccuracy of our thermodynamic integration. This suggests that the UV divergence remains identical to that of simple homopolymer melts, but this still needs to be confirmed.

The ODT boundaries in Figure 4, Figure 7, and Figure 10 were all determined by comparing ordered and disordered phases in identical simulation boxes, in which case the exact form of the UV divergence becomes irrelevant. This also remains true for comparisons between different sized simulation boxes provided their grid resolutions, $\Delta_{\alpha }$, are the same. However, this still represents a serious constraint, in large part because the efficiency of the Fourier transforms relies on $m_{\alpha }$ factorizing into small prime numbers [46]. We note that Delaney and Fredrickson [34] have avoided the UV divergence altogether by smearing the molecular interactions, which could likewise be implemented in FTS with the partial saddle-point approximation. In order to obtain universal results, the range of the interactions should be small relative to the width of the internal A/B interfaces. In addition though, the grid resolution needs to smaller than the range of the interactions, probably by a factor of at least four in order to adequately resolve the interaction profile. This implies that the 64×64×64 grid used to simulate the gyroid phase in Figure 5 would have to be switched to a 256×256×256 grid, which is currently unfeasible even on GPUs.

Another related issue is how to adjust the size of the simulation box to be commensurate with the equilibrium period of an ordered phase. For the lamellar and cylinder phases, the period can determined by minimizing the free energy with respect to the aspect ratio of the simulation box subject to a constant volume constraint [50,66]. Unfortunately, this strategy cannot be extended to triply-periodic phases. For the gyroid and Fddd phases in Figure 5 and Figure 6, respectively, the box size was adjusted to maximize the Bragg peaks, but this procedure is computationally expensive. The difficulty with using free energy is that changing the volume of the box affects the UV divergence. However, solving the previous problem of how to compare phases in boxes of different grid resolution would provide the means of solving this problem as well.

Now that field-theoretic simulations can handle large numbers of polymers with complicated architectures and multiple species [51,86,87,88,89], we expect them to gain widespread use. Hopefully, the open-source code provided in the Supplementary Materials will help facilitate this. The increased activity will undoubtedly spawn further developments that will expand its range of applications. As such, FTS is certain to become an invaluable complement to SCFT, which itself is one of the most successful theories in soft condensed matter physics [18].

## Supplementary Materials

The following are available online at https://www.mdpi.com/article/10.3390/polym13152437/s1, Open-source code for field-theoretic simulations of diblock copolymer melts (read SM.pdf for a description of the files).
