# Supplementary material for: Field-Theoretic Simulations for Block Copolymer Melts Using the Partial Saddle-Point Approximation
Source: Polymers (Basel). 2021 Jul 24;13(15):2437. doi: 10.3390/polym13152437 (PMC8347900; doi:10.3390/polym13152437)
Supplement: Supplementary file 1 [file polymers-13-02437-s001.zip › SM.pdf]

Supplementary Materials: Field-Theoretic Simulations for Block Copolymer Melts using the Partial Saddle-Point Approximation

Mark W. Matsen and Thomas M. Beardsley

Department of Chemical Engineering, Department of Physics & Astronomy, and Waterloo Institute for Nanotechnology, University of Waterloo, Waterloo, Ontario, N2L 3G1 Canada

Here we provide stripped-down versions of the FTS program for CPUs and GPUs with a sample input file corresponding to eight unit cells of the gyroid phase for $N_A = 36$, $N_B = 54$, and $\chi N = 14.3$ (i.e., $\chi_b N = 18.35$). We leave it to individual users to modify the code to their own tastes. The files are as follows:

fts.cc: The source-code for the CPU version of the program. Compilation requires the external library FFTW3 for fast Fourier transforms (www.fftw.org). The dependencies, random.h, and sim_eq.h, need to be present in the same directory as the source code. The executable file must be run in the same directory containing the input file.

fts.cu: The source-code for the GPU version of the program. Compilation requires the external library CUFFT for fast Fourier transforms (docs.nvidia.com). The dependencies, random.h, sim_eq.h, GPUerror.h, and GPUkernels.h need to be present in the same directory as the source code. The executable file must be run in the same directory containing the input file.

sim_eq.h: Required dependency for both the CPU and GPU source codes. It solves the simultaneous equations in the Anderson mixing algorithm; see Equation (32) in the main paper.

random.h: Required dependency for both the CPU and GPU source codes. It generates random numbers for $\mathcal{N}(\mu, \sigma)$; see Equation (36) in the main paper.

GPUerror.h: Required dependency for the GPU source code. Produces an error message in the event that the program fails.

GPUkernels.h: Required dependency for the GPU source code. Contains GPU subroutines for preparing $h_i(\mathbf{r})$, calculating $\phi_\pm(\mathbf{r})$, and performing general array operations.

input: The input file containing system parameters and the initial fields. The first line lists N, N_A, $\chi_b N$, $\sqrt{\bar{N}}$, and $\delta\tau N$, the second line lists m_x, m_y, m_z, L_x/R_0, L_y/R_0, and L_z/R_0, and the third line lists the number of equilibration steps, the number of simulation steps, and the frequency at which observables are sampled. The subsequent lines contain $NW_-(\mathbf{r})$ followed by $Nw_+(\mathbf{r})$ as one-dimensional arrays. The arrays are indexed by $r = (xm_x + y)m_y + z$, where the grid coordinates (x, y, z) take on values $x = 0$ to $m_x - 1$, $y = 0$ to $m_y - 1$, and $z = 0$ to $m_z - 1$.

output: An output file providing the system parameters and the final fields. The format is identical to that of the input file.

struct: An output file containing the spherically-averaged structure function. The lines contain pairs of kR_0 and $S(k)/\rho_0 N$ in sequential order of increasing wavevector, k.
